# Fine-grained multi-focus image fusion based on edge features

**DOI:** 10.1038/s41598-023-29584-y

**Published:** 2023-02-11

**Authors:** Bin Tian, Lichun Yang, Jianwu Dang

**Affiliations:** 1 College of Electronics and Information Engineering, Lanzhou Jiaotong University, Lanzhou, 730070 Gansu China; 2 Key Laboratory of Photoelectric Technology and Intelligent Control Ministry of Education, Lanzhou Jiaotong University, Lanzhou, 730070 Gansu China

**Keywords:** Computer science, Information technology

## Abstract

Multi-focus image fusion is a process of fusing multiple images of different focus areas into a total focus image, which has important application value. In view of the defects of the current fusion method in the detail information retention effect of the original image, a fusion architecture based on two stages is designed. In the training phase, combined with the polarized self-attention module and the DenseNet network structure, an encoder-decoder structure network is designed for image reconstruction tasks to enhance the original information retention ability of the model. In the fusion stage, combined with the encoded feature map, a fusion strategy based on edge feature map is designed for image fusion tasks to enhance the attention ability of detail information in the fusion process. Compared with 9 classical fusion algorithms, the proposed algorithm has achieved advanced fusion performance in both subjective and objective evaluations, and the fused image has better information retention effect on the original image.

## Introduction

Image fusion refers to the process of extracting the most meaningful information from multiple images of different sources and generating an image with more information and more beneficial to subsequent applications^1^. As a branch of image fusion, multi-focus image fusion has important research significance in the field of imaging. Limited by optical lenses, it is difficult to focus all objects with different depth of field^2^ when shooting by the camera. The main task of multi-focus image fusion is to fuse multiple images with different focus regions to get a full-focus image. At present, this technology has been widely used in robot vision, medicine, aerospace, military and other fields^3^.

According to the different research methods, image fusion algorithms can be divided into traditional fusion methods and deep learning-based methods. Since 1980s, Burt^4^ first applied Laplace Pyramid in the field of image fusion. Since then, pyramid decomposition methods such as low-pass pyramid, morphological pyramid and contrast pyramid^5^ have been applied to image fusion tasks. However, this method has some problems, such as image decomposition information redundancy, unable to extract the details of the direction subband, and the fusion effect is not ideal^6^. The use of wavelet transform^7^ can extract detail information in multiple directions, which is more beneficial to the preservation of detail information than pyramid transform. Since then, there have been many excellent image fusion algorithms, such as DCT^8^, NSCT^9^, DWT^10^, NSST^11^ and so on. GFF proposed by Li^12^ introduces the concepts of pixel saliency and image spatial continuity. And the guide filter is used to reconstruct the weight, which has a certain anti-noise ability. However, the saliency mapping generated by this method can not retain all the features of the image. MGFF proposed by Bavirisetti^13^ introduces the concept of multi-scale decomposition on the basis of guided filtering, which can find the visual significance of the whole scene from two images with different vision. However, due to the lack of selectivity to the information of each layer in the process of integration, the fused image is often prone to overlap blur and image brightness difference. Although the traditional fusion method has good interpretability, it is limited by the design of fusion rules, which to a certain extent limits the performance improvement of the traditional fusion method^14^.

In recent years, with the development of deep learning, with the strong ability of feature extraction and information reconstruction of neural network, it has been widely used in the field of image fusion. Liu^15^ introduced convolution neural network into the field of image fusion for the first time, and constructed a convolution neural network architecture based on Siamese structure for multi-focus image fusion task. The positive and negative sample pairs are constructed by Gaussian blurring of the original image. The convolution neural network is used to distinguish the clear pixels of the image block, and the final fusion task is realized through a series of post-processing operations. Compared with the traditional algorithm, the experiment has achieved remarkable results and has better fusion performance. However, the training cost of this method is high, and it takes millions of pixel blocks and thousands of iterations to achieve a good discriminant performance. Since then, Ram Prabhakar^16^ and others introduced unsupervised learning into the field of image fusion and proposed DeepFuse as a general image fusion framework. DeepFuse adopts the encoder-decoder structure and uses the structure similarity function as the loss function for image reconstruction. In the fusion stage, the encoder is directly used to encode and add the original image, and then passed into the decoder for decoding output to achieve the end-to-end image fusion. However, the fusion method of direct addition in DeepFuse is rough, so the fused image still retains the fuzzy information of the defocus region. For this reason, Li^17^ and others put forward the DenseFuse fusion task. DenseFuse uses DenseNet structure to design the encoder, which aims to retain the multi-scale information of the original image. At the same time, DenseFuse improved the fusion strategy, put forward the $L_{1}-norm$ fusion strategy, and achieved better results. DIFNet proposed by Hyungjoo Jung^18^ uses the eigenvector of the structure tensor to represent the direction of the maximum and minimum contrast of the multi-channel image and combines the image intensity information to construct a general fusion architecture. Although DIFNet is simple in structure. However, the fusion strategy of DIFNet is also rough, so the fusion image still has the defocus information of the original image, which can not maximize the effective information. SESF-Fuse proposed by Boyuan Ma^19^ considers the attributes of multi-focus images, designs a fusion strategy based on feature gradient, and achieves better discrimination performance. The SDNet proposed by Zhang^20^ constructs the fusion task directly from the gradient information and intensity information of the original image. Different from the traditional encoder-decoder structure, the result of SDNet coding is the fused image. The decoding process is the decoding process from the fused image to the original image. SDNet has achieved advanced fusion performance in a variety of fusion tasks, and has a good effect on preserving the texture details and luminance information of the original image. Although it is feasible to use the gradient information and intensity information of the image to consider the multi-focus fusion problem, it is not enough to consider these two features. Therefore, compared with the original image, the fused image has obvious color difference and a little loss of detail information. Starting from the image texture details, Zhang^21^ proposed MFF-GAN for multi-focus image fusion task. MFF-GAN uses the method of generating countermeasure network and self-supervised learning for image fusion, and achieves a better effect of texture detail retention, especially at the boundary of the focus region. However, it is precisely because MFF-GAN pays too much attention to the texture details of the image, resulting in less attention to the global information of the image, resulting in a lack of overall information retention of the fused image.

In order to improve the transfer ability of the fused image to the overall information and detail information of the original image, and to achieve better edge transition effect between the focused area and the defocused area. A two-stage image fusion algorithm based on fine-grained features and edge feature maps of the original image is proposed. Specific contributions are as follows.

- From the perspective of overall information coding of the original image, an encoder-decoder structure network based on DenseNet network and polarization self-attention module is designed. Compared with similar designs, it has better original information coding effect and lower reconstruction loss.
- From the perspective of pixel-by-pixel discrimination of the focused attributes of the encoded feature map, an image fusion strategy based on edge feature map is designed. Compared with the method of directly using the original image or the encoded feature map for fusion processing, it achieves lower discrimination error.

## Methods

### Overview of methods

**Figure 1:**
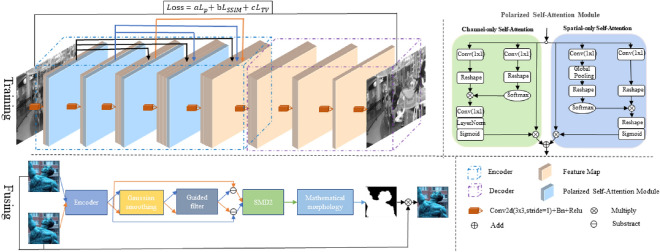
Fusion architecture diagram.

As shown in Fig. 1, the fusion algorithm is divided into two stages. In the training phase, we use the DenseNet network structure^22^ and the polarization self-attention module^23^(PSA) to construct the encoder-decoder structure for image reconstruction tasks. Among them, the use of DenseNet network structure is mainly to retain the multi-level information of the original image as much as possible. The polarization self-attention module is mainly to enhance the performance of the network on fine-grained pixel-level tasks. Under low computational overhead, the long-distance dependence of high-resolution input/output features is enhanced to estimate highly nonlinear pixel semantics. At the same time, we first randomly cut the original image into $64 \times 64$ image blocks and pass them into the model for training after a series of digital enhancement processing. This step is mainly to coordinate the PSA module to improve the model’s ability to focus on fine-grained information. In the fusion stage, the input of the model is the original image that does not need to be cropped. After using the trained encoder to encode the original image information, the encoded feature map is subjected to Gaussian smoothing and guided filtering respectively, and then cross-subtracted with the encoded feature map to obtain the corresponding edge feature map. Then, the gray variance product function is used to discriminate the focus area pixel by pixel, and the decision map is generated. After mathematical morphology processing, the final image fusion task is performed using a weighted method.

### Loss function

In this paper, the comprehensive loss function used by TransMEF^24^ is used, but the weight design of each loss function is reconsidered. (in view of the fact that TransMEF is mainly used for the consideration of multi-exposure fusion tasks) details are as follows.

1 $$\begin{aligned} L =aL_{p}+bL_{ssim}+cL_{TV} \end{aligned}$$

where $L_{p}$ represents represents the pixel loss function; $L_{ssim}$ represents the structural similarity loss function ; $L_{TV}$ represents the total variance loss function. a, b, c are the weight corresponding to each loss function. Different from TransMEF, this paper sets a, b, c as 1, 20, 1 respectively to enhance the model’s ability to pay attention to the structural similarity of reconstructed images in image reconstruction training tasks.

The $L_{p}$ loss function is used to measure the degree of pixel loss in the reconstruction task. The calculation method is as follows:

2 $$\begin{aligned} L_{p} = ||I_{out} - I_{in}||_{2} \end{aligned}$$

where $L_{out}$ and $L_{in}$ represent the input image and the reconstructed image, respectively, and $||\cdot ||_{2}$ represents the Euclidean distance between the them.

The $L_{ssim}$ loss function is used to measure the transfer of structural information in the reconstruction task. The calculation method is as follows:

3 $$\begin{aligned} L_{ssim} = 1-ssim(I_{out},I_{in}) \end{aligned}$$

$L_{TV}$ comes from VIFNet^25^. It is mainly used to consider the gradient information retention of the reconstructed image and further eliminate the influence of noise. It is calculated as follows:

4 $$\begin{aligned} R(p,q)= & {} I_{out}(p,q) - I_{in}(p,q) \end{aligned}$$

5 $$\begin{aligned} L_{TV}= & {} \sum _{p,q}(||R(p,q+1) - R(p,q)||_{2}+||R(p+1,q)-R(p,q)||_{2}) \end{aligned}$$

where *R*(*p*, *q*) represents the difference between the source image and the reconstructed image; p and q represent the horizontal and vertical coordinates of the image pixels, respectively.

### Fusion strategy

#### Decision diagram generation

In the fusion stage, a trained encoder is used to encode the fused image. Then, the encoded feature map is cross-subtracted with the original feature map using a Gaussian smoothing filter template with a size of $11 \times 11$ and guided filtering processing to obtain the corresponding edge feature map.The gray variance product function(SMD2) is used to determine the pixel level of image clarity according to the channel^26^.After mathematical morphology processing (A circular morphological operator is used here, and the threshold of $0.1\times H\times W$ is used for opening and closing operations to eliminate independent small areas), the final decision diagram is obtained.

6 $$\begin{aligned} f_{r}(x,y)= & {} \sqrt{\sum _{a=-r}^r\sum _{b=-r}^r{[F(x+a,y+b)-F(x+a+1,y+b)]}^2} \end{aligned}$$

7 $$\begin{aligned} f_{c}(x,y)= & {} \sqrt{\sum _{a=-r}^r\sum _{b=-r}^r{[F(x+a,y+b)-F(x+a,y+b+1)]}^2} \end{aligned}$$

8 $$\begin{aligned} F(x,y)= & {} \sqrt{\frac{|f_{r}(x,y) \parallel f_{c}(x,y)|}{(2r+1)^2}} \end{aligned}$$

$F_{r}(x,y)$ and $F_{c}(x,y)$ represent the row and column frequencies of the image, respectively.

Through the above method, the spatial frequency calculation results of images at different focusing positions are obtained respectively. The method of generating the initial weight map according to the frequency calculation results is shown in formula 9.

9 $$\begin{aligned} D(x,y) =\left\{ \begin{aligned} 1&\quad if\quad F_{1}(x,y) \ge F_{2}(x,y) \\ 0&\quad otherwise \\ \end{aligned} \right. \end{aligned}$$

#### Weight fusion

According to the generated final decision map, the final image fusion is performed in a weighted manner, and the calculation method is shown in Formula 10.

10 $$\begin{aligned} F(i,j) = D(i,j)A(i,j)+(1-D(i,j))B(i,j) \end{aligned}$$

### Fusion evaluation indicators

So far, there is no evaluation method that can be applied to all fusion algorithms. Therefore, subjective evaluation and objective evaluation indexes are used to evaluate the fused image from two aspects. In the objective evaluation index, MI(mutual information)^27^, SSIM(structural similarity)^28^, PSNR(peak signal to noise ratio)^29^, AG(evaluation gradient)^30^, SF(spatial frequency)^31^, $Q^{MI}$^32^ and$Q^{AB/F}$^33^ were used to evaluate the fused image. The specific explanation is as follows.

MI(Mutual Information)

Mutual information represents how much information of the source image is transferred to the fused image. The larger the mutual information value, the better the image fusion effect.

SSIM(structural similarity)

SSIM is used to measure the structural similarity between the fused image and the source image. The larger the SSIM value, the more similar the structure of the fused image to the structure of the source image.

PSNR(peak signal-to-noise ratio)

The peak signal-to-noise ratio is used to measure the ratio between the effective information of the image and the noise, which can reflect whether the image is distorted. The greater the PSNR, the better the image quality.

AG(average gradient)

The average gradient can reflect the ability of the image to express the contrast of small details, and also reflect the characteristics of texture transformation in the image. Therefore, the greater the average gradient, the clearer the image.

SF(Spatial Frequency)

Spatial frequency reflects the overall activity of the image in the spatial domain, that is, the change rate of image gray. The larger the spatial frequency, the better the quality of fused image.

$$Q^{MI}$$

$Q^{MI}$eliminates the impact of information entropy on the fusion image quality assessment results, can better measure the amount of information transfer between the original image and the fused image. The larger the $Q^{MI}$, the better the image fusion.

$$Q^{AB/F}$$

$Q^{AB/F}$ can better reflect how much edge information in the source image is transmitted to the fused image. The greater the value of $Q^{AB/F}$ indicates that the fused image retains more edge information of the source image and the fusion effect is better.

## Experiments

### Experimental settings

In this paper, 10,000 images from the MS-COCO2014 data set^34^ are selected for training, and 38 pairs of public multi-focus images^35,36^ are selected for test evaluation. The images used in the training phase were all processed with data enhancement (randomly cut to $64 \times 64$ size, randomly rotated, and randomly transformed in brightness, contrast and tone). The batchSize selected in this paper is 32, the learning rate is $1\times 10^{-4}$, and the number of iterations is 50. AdamW optimizer and Warm up learning rate strategy are used for model training.

The hardware platforms used in the experiment are NVIDIA RTX3080 GPU, 12th Gen Intel(R) Core(TM) i7-12700KF CPU and 32GB memory. The software platform used is Pycharm integrated development environment. The programming language used is Python and Pytorch, Skimage and OpenCV libraries are mainly used for experiments.

### Image fusion evaluation

In order to evaluate the performance of the fusion algorithm in this paper, we choose GFF^12^ and MGFF^13^ based on traditional fusion strategy, and end-to-end DeepFuse^16^, DenseFuse^17^, DIFNet^18^, SDNet^20^ and MFF-GAN^21^ based on deep learning. Nine algorithms, CNN^15^ and SESF-Fuse^19^, which need post-processing operation, are compared with this algorithm by subjective and objective evaluation indexes, as follows.

#### Subjective evaluation

**Figure 2:**
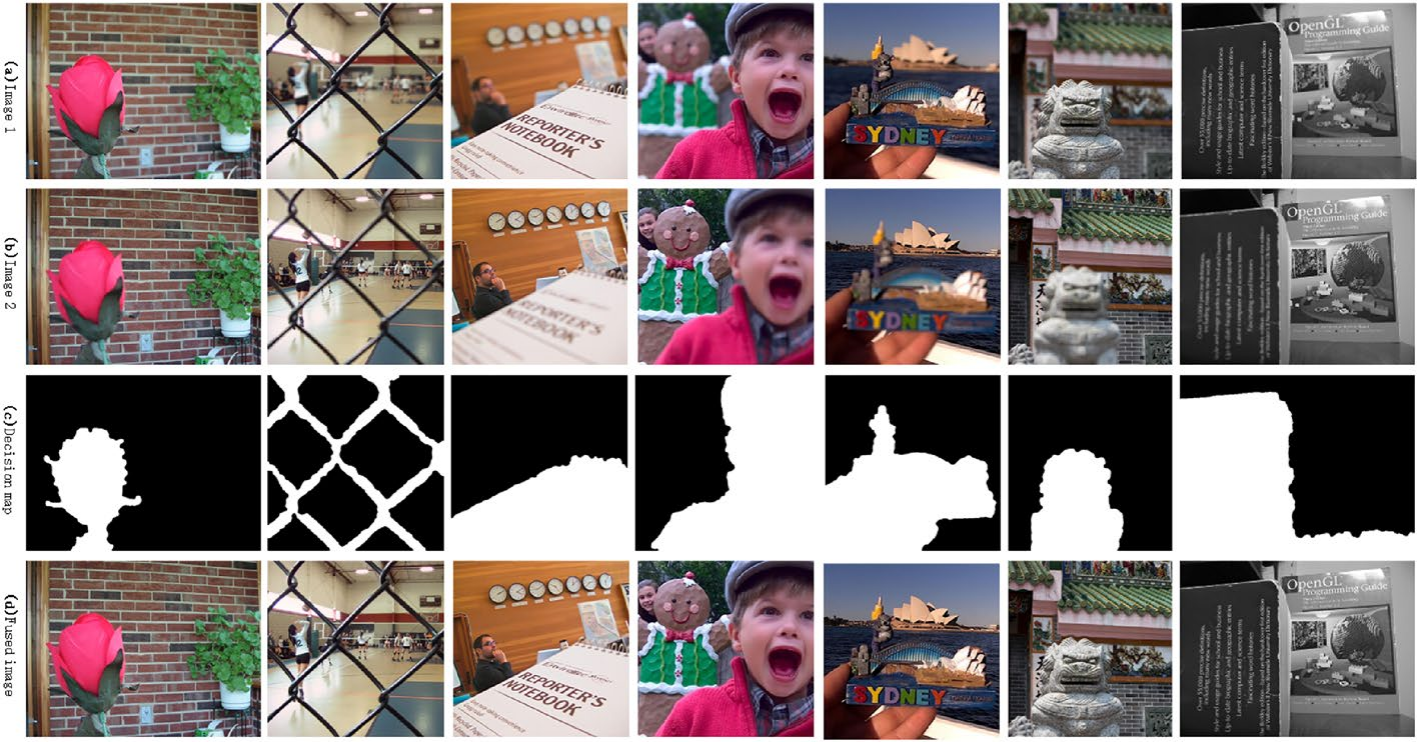
Fusion image results^35,36^.

Using this algorithm for image fusion, visualization 7 pairs of images before and after fusion and the corresponding decision map results are shown in Fig. 2.

From Fig. 2, we can see that the clear and non-clear areas of the focused image can be accurately identified by this algorithm, and the fused image is more clear subjectively. In order to further analyze the fusion performance of this algorithm compared with other algorithms. Here, several different fusion images are selected to compare the sharpness of the fused local image and the difference between the fused image and the original image.

**Figure 3:**
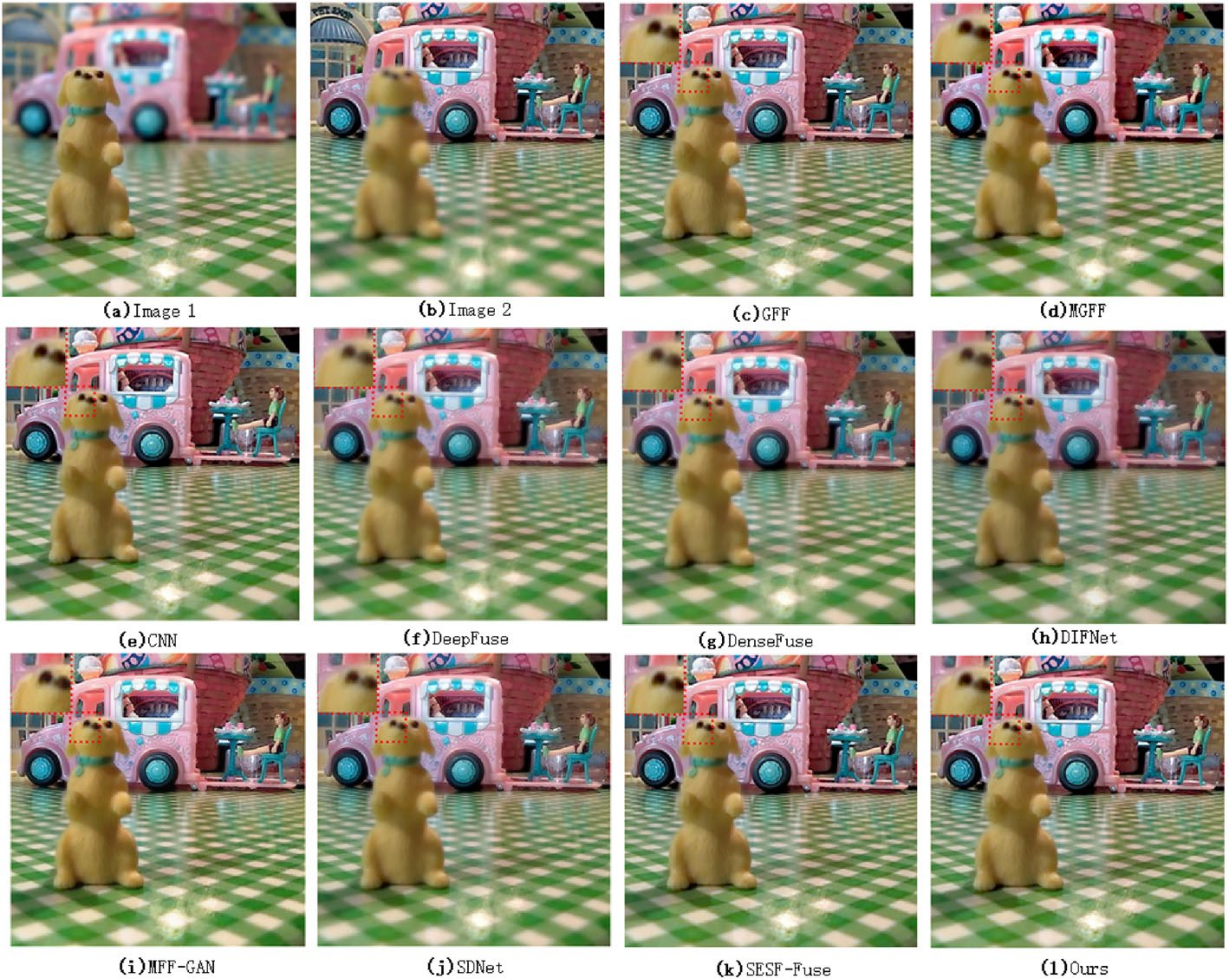
Image local comparison 1(Lytro 17^35^).

**Figure 4:**
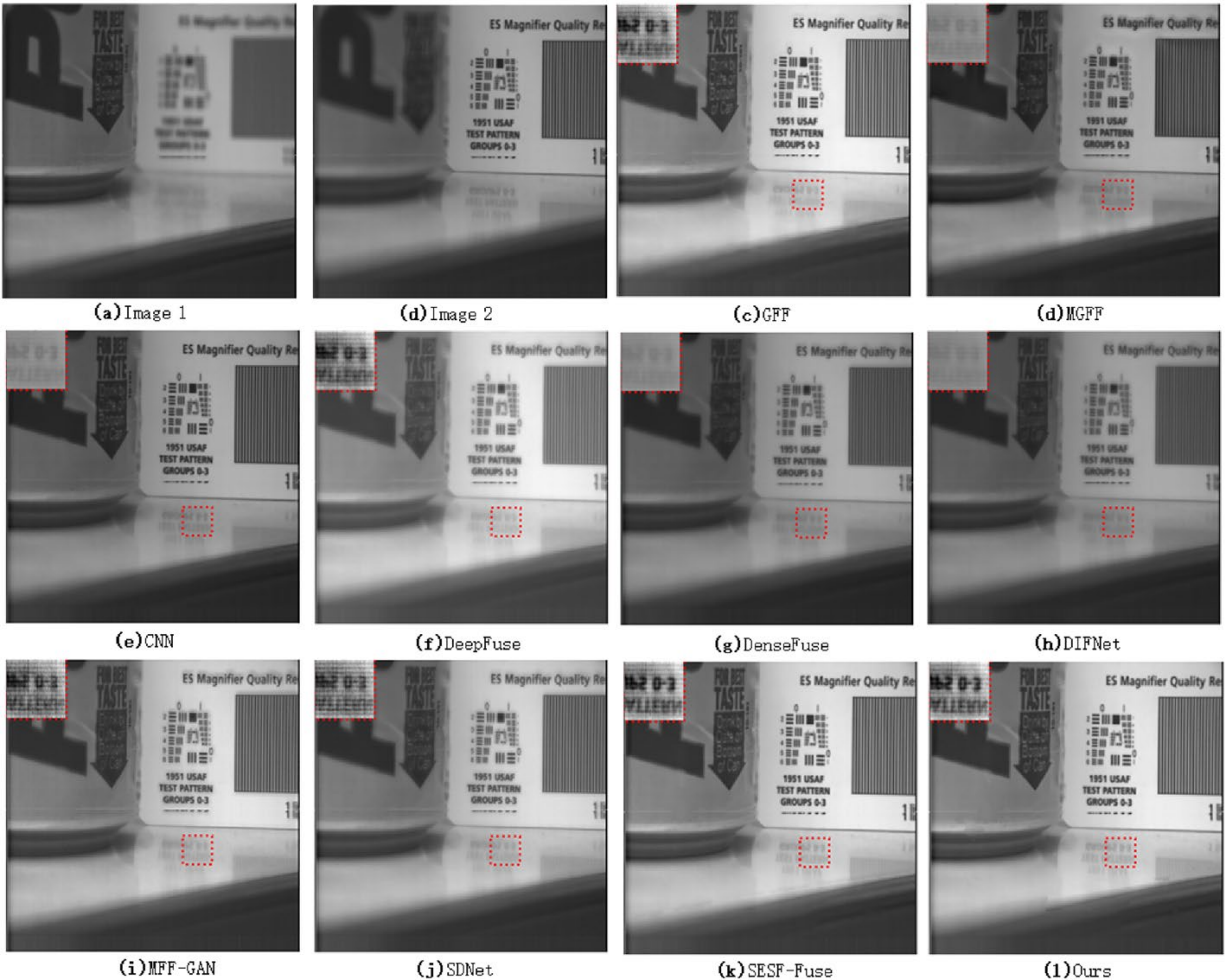
Image local comparison 2(Savic pepsi^36^).

The local magnification comparison result of the fused image is shown in Figs. 3, 4.

From the local magnification images of the fused images of various algorithms in Fig. 3 (the red marked area in the upper left corner of each image), we can see that there is obvious blur in the fused images using DeepFuse, DenseFuse, DIFNet and SDNet algorithms for multi-focus image fusion. GFF, MGFF, CNN, MFF-GAN, SESF and the algorithm in this paper are relatively good. The reason is that both DeepFuse and DenseFuse use a simple coding feature graph addition method to carry out the fusion task, and the final fused image is obtained after decoding by the decoder. This fusion method can not take into account the difference information between the original images, and only plays a comprehensive role in the information of the original images, which weakens the difference information between the original images to a certain extent. DIFNet uses concat for image fusion also has the same problem, so the fused image still retains the information of the defocus region of the original image. SDNet introduces the gradient information and intensity information of the image to consider the fusion image. Compared with DeepFuse and DenseFuse, the fusion effect is relatively better, but there is still some loss of detail information, so the fusion image still has some fuzziness.

As can be seen from Fig. 4, the loss of detail information of the fused image using MGFF, CNN, DenseFuse and DIFNet algorithms is still obvious. Although the GFF, DenseFuse, MFF-GAN and SDNet algorithms have the ability to retain the detail information, the detail information of the fused image is still slightly weakened. The second line of text in the local magnified image in the upper left corner of the fusion image corresponding to the algorithm is particularly blurred, and the specific letters can not be distinguished, but the image fused by SESF algorithm and this algorithm can still recognize the specific text content.

**Figure 5:**
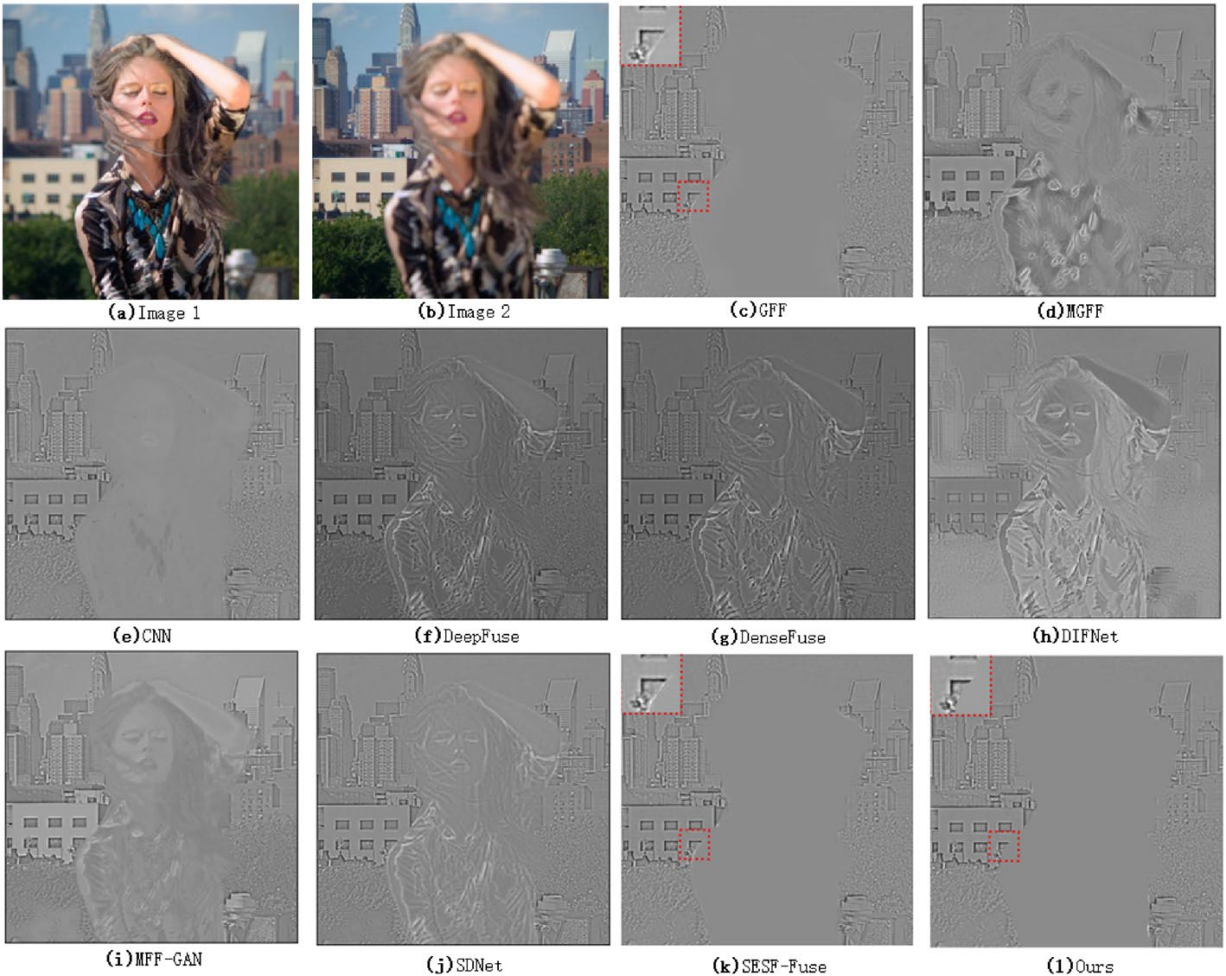
Comparison of difference between fused image and original image 1(Lytro 7^35^).

**Figure 6:**
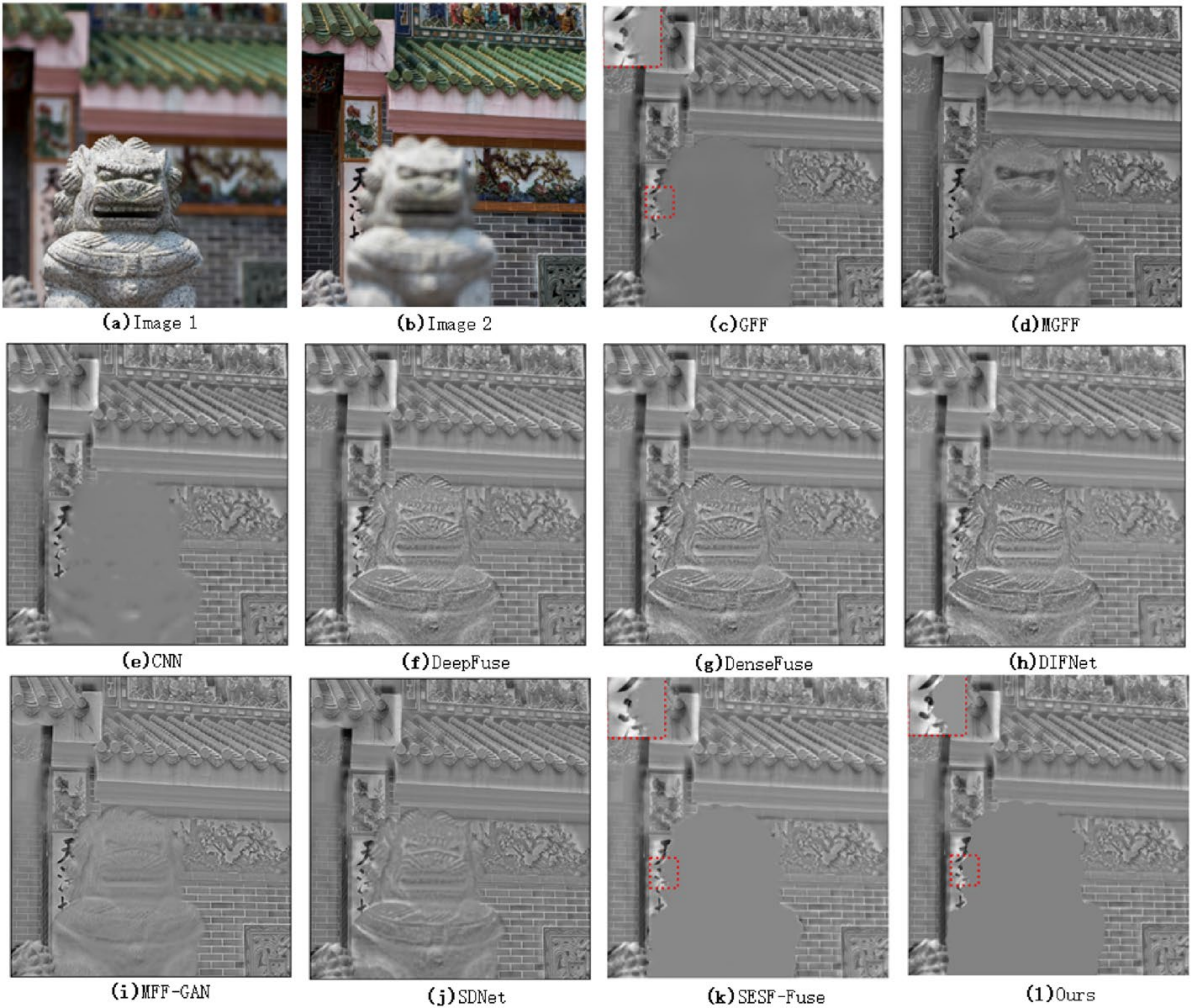
Comparison of difference between fused image and original image 2(Savic temple^36^).

In addition, in order to more accurately measure the original information loss of the fused image after using various algorithms for multi-focus image fusion, it is considered from the point of view of the difference map (the difference between the fused image and the original image). The specific results are shown in Figs. 5, 6.

As can be seen from Fig. 5, the information loss in the focus region of the original image is more obvious in the image fused with MGFF, DeepFuse, DenseFuse and DIFNet algorithms. There are also a lot of information loss in MFF-GAN and SDNet algorithms. Although the retention effect of CNN is better than the above algorithm, there is still a little loss of original information. In comparison, GFF, SESF and the algorithm proposed in this paper are better in preserving the overall information of the focus region of the original image.

However, as far as the boundary of the focus region of the image is concerned, from the local magnification of the focus boundary of the GFF and SESF algorithms in Fig. 6, we can see that the focus boundary of the image fused by GFF and SESF algorithms has a little incomplete phenomenon. It is worth noting that the SESF algorithm is less incomplete than the GFF algorithm. The reason is that the GFF algorithm is the focus attribute discrimination directly on the original image, while the SESF algorithm is the focus attribute discrimination task on the corresponding feature image after encoding the original image. This shows that the feature coding of the original image can reduce the noise of the original image and reduce the incomplete degree of the focus edge. This algorithm adopts the same idea as SESF, first encodes the feature, and then discriminates the focus attribute on the encoded feature graph. At the same time, this algorithm adds the edge feature extraction processing of the coded feature graph and the focus attribute discrimination task combined with the edge feature information of the encoded feature graph. It can be seen that the focus boundary of the fusion image using this algorithm is relatively smooth, and there is no obvious incomplete phenomenon, which also proves the rationality of the method proposed in this paper.

According to the analysis of the retention effect of the image detail information in the local magnified image in the subjective evaluation and the overall information in the focus region of the original image after fusion in the difference map, the performance of this algorithm is better than that of other algorithms.

#### Objective evaluation

In order to quantify the fusion effect of various algorithms, MI, SSIM, PSNR, AG, SF, $Q^{MI}$ and $Q^{AB/F}$ have a total of 7 objective evaluation indicators compared the combined image, and the results are shown in Table 1.

**Table 1:** Comparison of various algorithms.

Algorithms	Index evaluation						
	MI	SSIM	PSNR	AG	SF	$Q^{MI}$	$Q^{AB/F}$
GFF	7.1875	0.7951	26.0317	8.2364	18.9739	0.9798	0.4499
MGFF	5.2626	0.8228	24.9116	7.2243	17.1650	0.7170	0.3933
CNN	6.0823	0.8099	25.8275	*8.4265*	19.2010	0.8399	0.4431
DeepFuse	5.7598	*0.8503*	26.2962	5.4042	12.0751	0.7841	0.3460
DenseFuse	5.7582	**0.8570**	**28.2387**	5.1400	11.4805	0.7889	0.3360
DIFNet	5.5582	0.8428	25.4697	4.7213	10.5981	0.7682	0.2604
MFF-GAN	5.4468	0.8116	24.7821	**8.6212**	**19.8732**	0.7432	0.3779
SDNet	5.6940	0.8339	*26.3376*	7.5673	17.4520	0.7745	0.3905
SESF	*8.1600*	0.8058	26.2059	8.3985	*19.2819*	*1.1115*	*0.4643*
Ours	**8.4180**	0.8054	26.2222	8.4013	19.2458	**1.1469**	**0.4664**

Bold is the best, italic is the second best.

As can be seen from Table 1, among the seven fusion indexes, this algorithm achieves 3 optimal fusion performance, which is better than the other 9 fusion algorithms. Compared with the GFF and MGFF algorithms based on the traditional fusion strategy, this algorithm surpasses all the indexes in an all-round way. Compared with the DeepFuse, DenseFuse, DIFNet, MFF-GAN and SDNet algorithms based on deep learning end-to-end fusion network, this algorithm is better than them in 6, 5, 7, 4 and 6 indexes, respectively. Compared with the CNN and SESF algorithms based on the need post-processing operation, the algorithm in this paper has 7 and 5 indexes that exceed them respectively.

To sum up, combined with the results of subjective evaluation and objective evaluation, we can see that this algorithm has a better ability to retain the original information than other algorithms in multi-focus fusion tasks. On the whole, better fusion performance has been achieved.

#### Fusion time evaluation

In order to meet the needs of practical applications, we increase the analysis of the fusion time of each algorithm. Fusion experiments were performed on 38 pairs of public test fusion datasets. Considering that the initial running algorithm is affected by factors such as resource loading, this will bring an increase in time. At the same time, the fusion time of different images is not the same. Therefore, we ran each algorithm 11 times and calculated the duration from the second time. The final fusion duration is the average of 10 calculations, as shown in Table 2.

**Table 2:** Comparison of fusion time of various algorithms.

Algorithms	GFF	MGFF	CNN	DeepFuse	DenseFuse
Times(s)	0.9256	3.7951	220.2890	0.1756	**0.0318**

Algorithms	DIFNet	MFF-GAN	SDNet	SESF	Ours
Times(s)	0.2808	0.1935	*0.1415*	0.1991	0.2834

Bold is the best, italic is the second best.

As can be seen from Table 2, although the proposed algorithm improves the discrimination accuracy by analyzing the edge features of the encoded feature map to distinguish the focus area of the image, it also brings an increase in the duration. Compared with SESF, which needs post-processing, the time of image fusion algorithm is increased by about 0.08 s, which is acceptable. It is worth mentioning that the fusion time of each algorithm is only a relative concept and cannot be generalized. Because the fusion time will be significantly different due to the programming language and whether the code optimization is performed. For example, the original DenseFuse algorithm written in Tensorflow does not have such a fast fusion rate, but the original author achieved a faster fusion rate after using Pytorch. The length of CNN ’s fusion time is also debatable, because CNN is written in Matlab language, so he cannot use CUDA to accelerate calculations like Tensorflow or Pytroch code^37^.

### Ablation experiment

This paper explores the loss function, model construction and fusion strategy. In order to verify the effectiveness of each research, a confirmatory analysis is carried out from the above three aspects, as follows.

#### Loss function analysis

In the task of image reconstruction, this paper uses the comprehensive loss function in TransMEF^24^ and adjusts its weight. The effects of each loss function are analyzed here, and the mean and variance results of the reconstruction loss ($L_{p}+20L_{ssim}+L_{TV}$) are shown in Table 3.

**Table 3:** Comparison of loss functions.

Reconstruction loss	$L_{p}+20L_{ssim}+L_{TV}$	$20L_{p}+L_{ssim}+20L_{TV}$	$L_{p}+1000L_{ssim}$
Mean	**0.1304**	0.8373	0.4473
STD	**0.1519**	0.9499	0.5854

Significant values are in bold.

It can be seen from Table 3 that structural similarity is more important than pixel loss in image reconstruction tasks. Therefore, the effect is better when the weight of structural similarity loss is greater than that of pixel loss. In addition, it can be seen that the performance of the loss function used in this paper is also better than that of the loss function($L_{p} + 1000L_{ssim}$) commonly used in traditional image fusion tasks, which again proves that the introduction of Ltv loss has the effect of reducing image noise. Therefore, on the whole, the loss function used in this paper is reasonable.

#### Attention mechanism analysis

In the task of image reconstruction, multiple attention mechanisms are used to build the model, and the corresponding model reconstruction loss is shown in Table 4.

**Table 4:** Performance comparison of various attention mechanisms.

Reconstruction loss	PSA	CBAM^38^	SENet^39^	GCNet^40^
Mean	**0.1304**	0.2371	0.1653	0.1923
STD	0.1519	0.4118	0.2451	**0.1152**

Significant values are in bold.

From the reconstruction loss results of the original image encoding and decoding process using various attention mechanisms in Table 4, we can see that using PSA to build the model has less reconstruction loss than other attention mechanisms. The reason is that CBAM pays attention to the image information by the combination of space and channel. However, this is often goal-based, and is better at paying attention to local information, and the ability to pay attention to global information is insufficient. The way that SENet uses global context to calibrate the weights of different channels to adjust channel dependencies does not make full use of global context information. Although GCNet has stronger global attention ability, its attention effect has not been obviously reflected in this task for pixel blocks with a size of $64 \times 64$ after clipping. PSA combines the advantages of both CBAM channel attention mechanism and spatial attention mechanism, and integrates the global modeling ability of self-attention mechanism, which can improve the model’s ability to pay attention to fine-grained pixels without increasing the computational complexity. Therefore, PSA has achieved better results in the task of this article.

#### Fusion strategy analysiss

**Figure 7:**
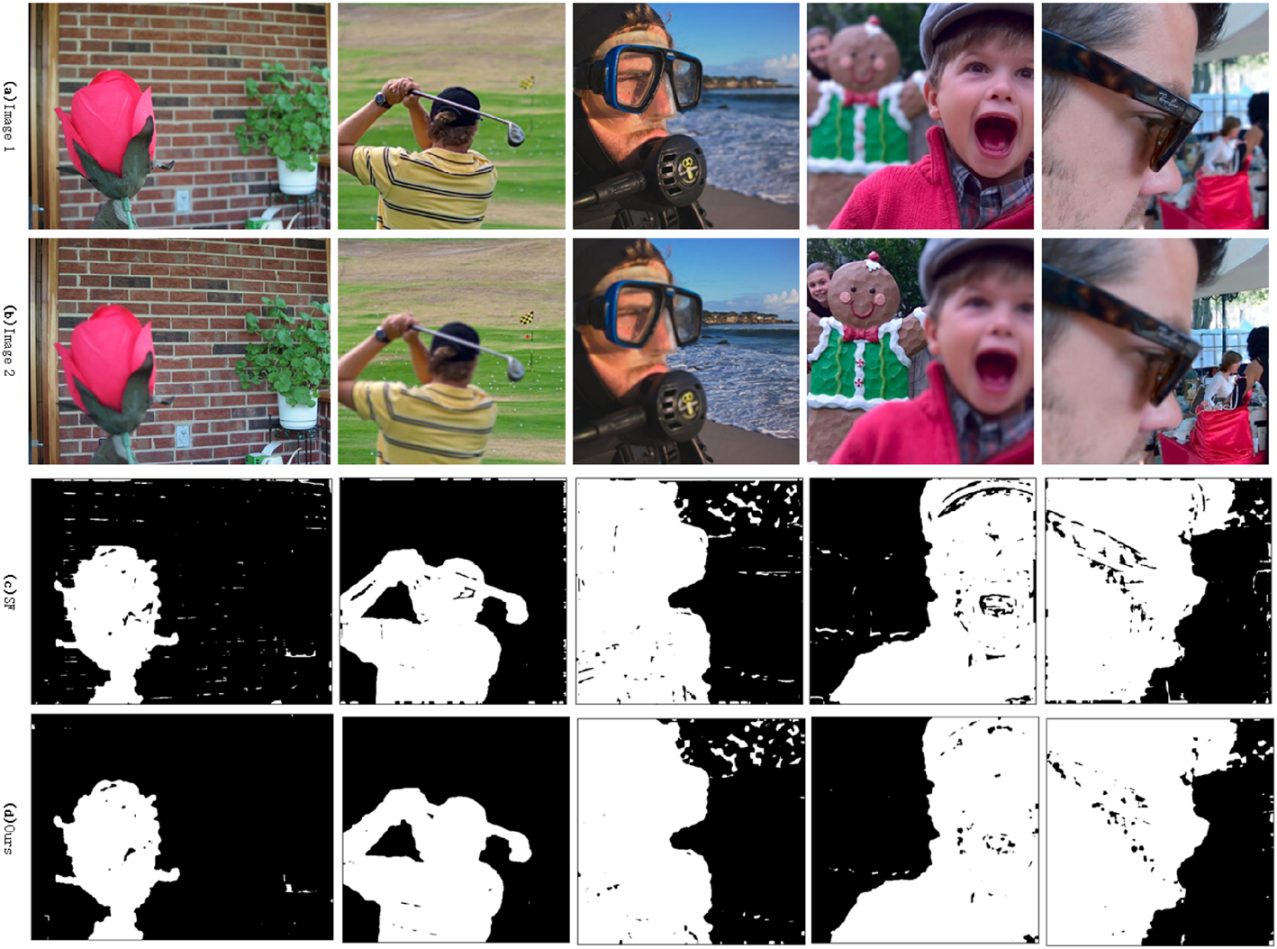
Comparison of fusion strategies^35,36^.

In the image fusion task, an image fusion strategy based on edge feature graph is proposed in this paper. Without using mathematical morphology processing, it is compared with the SF (spatial frequency) fusion strategy used by SESF, and the result is shown in Fig. 7.

It can be seen from Fig. 7 that the fusion strategy based on edge feature map proposed in this paper is used for image fusion task. In the process of pixel-level clear pixel discrimination, the discrimination accuracy is higher. The reason is that the discrimination method based on spatial frequency is mainly based on the gradient information of the encoded feature map. For feature maps with noise interference, it often leads to inaccurate discrimination. The discrimination method based on edge feature map is preprocessed by Gaussian filtering and guided filtering, which reduces the noise information of the original image and increases the gradient information of the original image to a certain extent, so this method has achieved good discrimination effect. Overall, the fusion strategy proposed in this paper is effective.

## Conclusion

This paper combines DenseNet network structure and polarization self-attention module to design an image reconstruction network based on unsupervised learning, which achieves better information retention. At the same time, we focus on increasing the consideration of the design of multi-focus image fusion strategy, and propose a fusion strategy based on edge feature map for image fusion task, which achieves better image focusing attribute discrimination effect. In comparison with a variety of algorithms, the proposed algorithm has achieved priority fusion performance in both subjective evaluation and objective evaluation. However, it is also found that there are still some discrimination errors in the way of using edge feature map information. How to further improve the accuracy of focus attribute discrimination of image fusion is our future research direction. In addition, due to the blurring of the focus boundary of the original image, the use of decision maps for fusion will cause the fused image to have a certain gradient dispersion phenomenon at the decision boundary. How to further improve the performance of feature extraction and reduce the occurrence of gradient dispersion remains to be further studied.

## Data Availability

The tralning sets that support the findings of this study are available at https://cocodataset.org/#download. The test sets that support the findings of this study are available at https://dsp.etfbl.net/mif/ and https://github.com/xingchenzhang/MFIF/tree/main/input. In addition, all data related to this experiment can be obtained by contacting this email(Jianwu Dang: ylc1377759045@163.com).
